# Tertiary lymphoid structures in the era of cancer therapy

**DOI:** 10.1186/s13046-026-03644-3

**Published:** 2026-02-11

**Authors:** Isaias Hernández-Verdin, Anna Dimberg, Daniela S. Thommen, Camilla Engblom, Lucile Vanhersecke, Karina Silina, Tullia C. Bruno, Wolf H. Fridman, Paola Nisticò, Catherine Sautès-Fridman

**Affiliations:** 1 Centre de Recherche des Cordeliers, Sorbonne Université, INSERM, Université de Paris, Paris, 75006 France; 2 Department of Immunology, Genetics and Pathology, Science for Life Laboratory, The Rudbeck Laboratory, Uppsala University, Uppsala, Sweden; 3 Division of Molecular Oncology and Immunology, Oncode Institute, Netherlands Cancer Institute, Amsterdam, The Netherlands; 4 Division of Immunology and Respiratory Medicine, Department of Medicine Solna, Karolinska Institutet, Science for Life Laboratory, Stockholm and Center for Molecular Medicine, Karolinska University Hospital, Stockholm, Sweden; 5 Departement de Biopathologie, Institut Bergonié, Bordeaux, France; 6 Institute of Pharmaceutical Sciences, Swiss Federal Institute of Technology, Zurich, Switzerland; 7 Department of Immunology, University of Pittsburgh, Pittsburgh, PA 15213 USA; 8 Tumor Microenvironment Center, Hillman Cancer Center, University of Pittsburgh, Pittsburgh, PA 15213 USA; 9 Tumor Immunology and Immunotherapy Unit, IRCCS Regina Elena National Cancer Institute, Rome, Italy

**Keywords:** Tertiary lymphoid structures, Tumor microenvironment, Immunotherapy, Heterogeneity, Spatial multi-omics

## Abstract

On September 9th, 2025, the 3rd Workshop on Tertiary lymphoid structures (TLS) took place in Utrecht as pre-meeting to CICON2025. Advances in TLS biology, diversity, and clinical significance are rapidly reshaping the field. The studies discussed here reveal the heterogeneity in TLS architecture, cellular composition, functional states, and developmental trajectories, shaped by tumor-specific chemokine gradients. Spatial multi-omics revealed that TLS appearing histologically similar can contain T cells with distinct functional profiles, influencing clinical outcomes independently of conventional immune markers. Efforts toward TLS standardization are gaining attraction through pathology-based algorithms capable of reliably identifying mature TLS across cancer types, supporting reproducible stratification and prediction of immunotherapy response. However, challenges remain in glioblastoma and ovarian cancer, where TLS are rare, anatomically constrained, and strongly influenced by local tissue niches. Experimental models demonstrate that vascular-targeted cytokine delivery can induce TLS formation and promote T cell–dependent tumor control, supporting novel therapeutic avenues. Technological advances, including spatially resolved antigen receptor sequencing, now allow high-resolution mapping of B- and T-cell clonotypes, clonal evolution, and early antigen discovery within TLS. Across cancers, TLS enriched in activated B cells, memory B cells, and plasma cells emerge as key drivers of sustained immune activation. Spatial analyses also reveal interactions between TLS and specific fibroblast or mesenchymal subsets that modulate immunotherapy response. Finally, integrative studies identify tumor-intrinsic metabolic pathways, such as GABA production, that suppress TLS activity and contribute to immunotherapy resistance. Collectively, these findings establish TLS as dynamic, spatially organized immune hubs with broad implications for cancer prognosis and therapy.

## 50 shades of lymphoid structures in cancer

Karina Silina (Group leader, IPW D-CHAB ETH Zurich, Switzerland)

In her opening presentation at the tertiary lymphoid structures (TLS) symposium, Karina Silina discussed the multifaceted heterogeneity of TLS from structural, compositional, functional, and developmental perspectives. Dr Silina presented recent unpublished data revealing that distinct dynamics of chemokine gradients accompany TLS development in different tumor types. Further, her data demonstrated that T cell functionality differentiates histologically indistinguishable TLS in lung cancer patients with divergent clinical outcomes. These findings were obtained through spatial omics analyses at both the transcriptomic and proteomic levels.

**Fig. 1:**
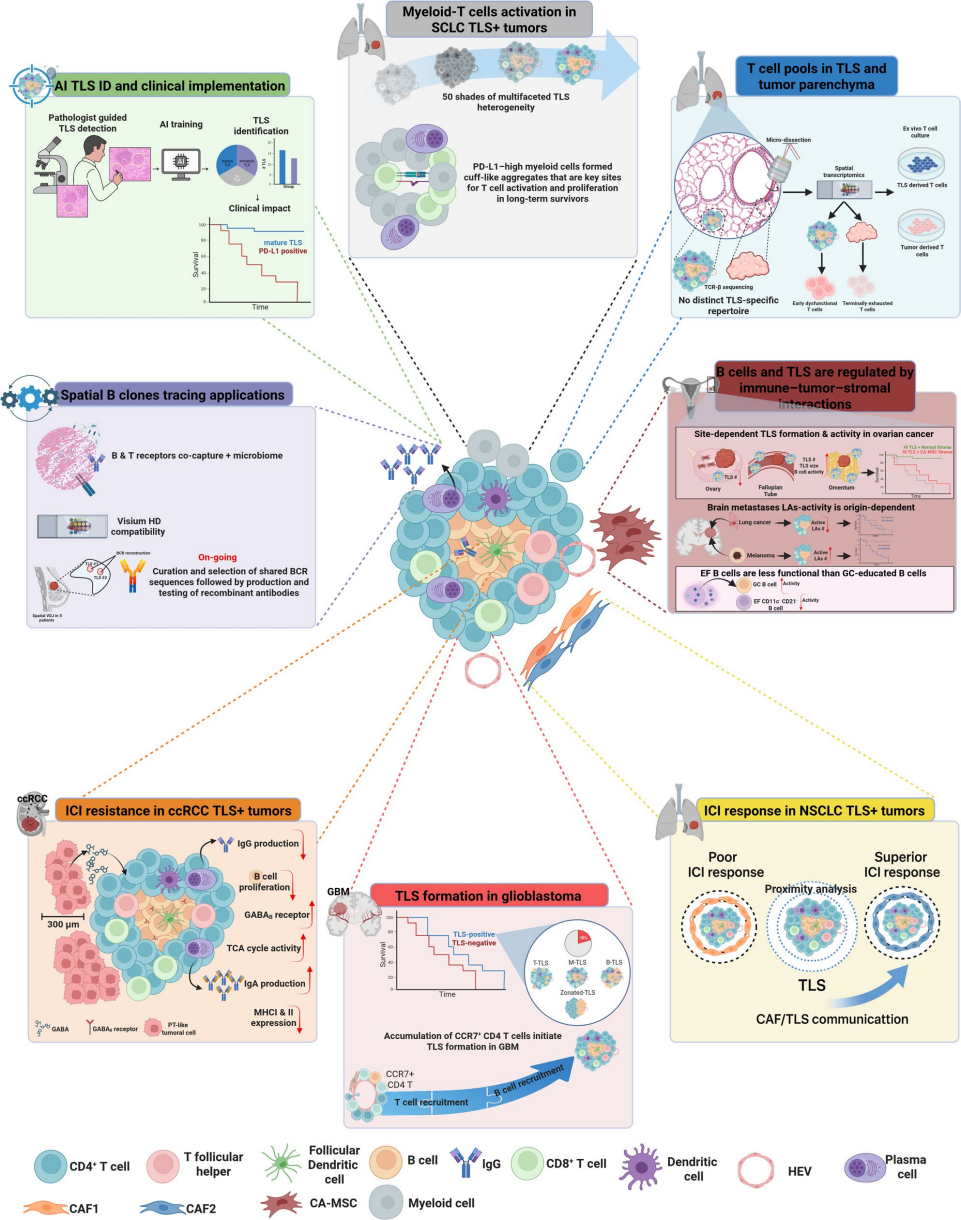
Graphical abstract of the eight different studies (represented in each colored panel) presented in the 3rd workshop on TLS during CICON2025 pre-meeting. AI, artificial intelligence; TLS, tertiary lymphoid structures; SCLC, small cell lung cancer; ID, identification; NSCLC, non-small cell lung cancer; BCR, B cell receptor; TCR, T cell receptor; PT, proximal tubule; GBM, glioblastoma; T-TLS, T cell rich TLS; M-TLS, mixed TLS; B-TLS, B cell rich TLS; CAF, cancer associated fibroblasts. CAF1 is associated with poor ICI resopnse in NSCLC TLS + tumors; while CAF2 with superior ICI response. ICI, immune checkpoint inhibitors; LAs, lymphoid aggregates; EF, extrafollicular; GC, germinal center

Finally, Dr Silina underscored the importance of myeloid cell niches in shaping the tumor immune microenvironment. In highly inflamed, TLS-rich lung tumors from long-term survivors, PD-L1-high myeloid cells formed cuff-like aggregates surrounding tumor nests in association with plasma cells and T cells. These cuffs represented key sites of T cell activation and proliferation and were notably absent in similarly inflamed TLS-rich tumors from short-term survivors (Fig. 1, gray panel).

## A standardized pathology for TLS screening

Lucile Vanhersecke (Pathologist, Institut Bergonié, Bordeaux, France)

Lucile Vanhersecke presented a pathology screening algorithm for mature TLS identification [1] based on Hematoxylin, Eosin and Saffron (HES) staining associated with CD20/CD23 immunostainings to detect germinal centers with a CD23 + follicular dendritic cells (FDC) network. This screening method was applied in a pan-cancer TLS study [2] and in the PEMBROSARC clinical trial to screen for TLS positive patients [3]. TLS were defined as lymphoid aggregates displaying a CD20^+^B-cell zone juxtaposing a CD20^-^ T-cell area, containing ≥ 50 cells, located intratumorally or ≤ 1 mm from the tumor front. Dr Vanhersecke defined a TLS as mature (mTLS) when a germinal center (GC) was visible on HES, or when CD23^+^FDC were identified in B cell follicles. She discussed critical considerations for this assay, including distinction of CD23^+^ FDC from CD23^+^ mature B cells, representative block selection and exclusion of secondary lymphoid organs (e.g. MALT or LN).

In the retrospective pan-cancer cohort, mature TLS independently predicted improved immunotherapy response and survival regardless of PD-L1 or CD8⁺ T-cell status, and TLS predictive value for PD-1 blockade was prospectively validated in the PEMBROSARC clinical trial by significantly higher objective response and 6-month non-progression rates in TLS-positive patients versus unselected controls.

Standardized TLS screening thus provides robust clinical decision-making support for immunotherapy, though limitations including labor intensity, tissue consumption, and scalability challenges persist. Dr Vanhersecke’s ongoing work explores deep learning development for TLS assessment to enable broad-scale clinical implementation (Fig. 1, green panel).

## Cellular and molecular regulation of TLS formation in glioblastoma

Anna Dimberg (Professor, group leader, Uppsala University, Uppsala, Sweden)

Anna Dimberg shared her work focusing on the characterization of TLS in human glioblastoma and on induction of these structures in murine glioma models. TLS are rare in glioblastoma, are often localized in proximity to meningeal tissues or ventricles and are less commonly present within the tumor tissue [4]. The morphology of these structures is influenced by their anatomic location, and those that reside close to the skull are often markedly elongated. The presence of TLS is associated with a higher T cell infiltration in human glioma and prolonged survival, but is only observed in 15% of glioblastoma patients. Consistent with this, previous data from the group have shown that TLS-induction using a vascular-targeted adeno-associated viral (AAV) coding for the cytokine LIGHT triggers T-cell-dependent anti-tumor immunity and prolongs survival in murine glioma models [5].

TLS are heterogeneous in human glioblastoma, and include T-cell rich, mixed, B-cell rich and clearly zonated structures. In murine models, a vast majority of TLS are T-cell rich early during glioma development, while B-cell rich structures dominate at late stages. Combined evidence provided by single-cell RNA sequencing (scRNA-seq) and spatial transcriptomics of human glioblastoma and functional studies in experimental models suggest that accumulation of CCR7^+^ CD4 T cells initiates TLS formation in glioblastoma, followed by activation of lymphoid tissue organizer cells and B-cell recruitment [6] (Fig. 1, red panel).

## Mapping antigen receptors in human tumors… and beyond

Camilla engblom (Assistant Professor, Karolinska Institute, Stockholm, Sweden)

Understanding which B and T cell clonotypes drive anti-tumoral responses and their specificity is a key issue in TLS biology. Camilla Engblom presented a spatial transcriptomics-based method she developed to capture B and T cell receptor sequences in human tissues [7]. Dr Engblom presented on-going work to extend the Spatial VDJ methodology, including to murine tissues, co-capture of B and T cell clonotypes with microbes, and the first steps towards a high resolution Visium HD 3’-based version. Application of the Spatial VDJ method to multi-regional human breast tumors (*n* = 36 Visium/Spatial VDJ libraries across five patients) allowed her to resolve B and T cell clonal diversity, capture B cell clonal evolution, and detect extensive clonal sharing and infiltration into TLS and tumor areas that varied within the same patient tumor. To survey antigen specificity, she also produced and tested recombinant antibodies from ten selected B cell receptor sequences from two breast cancer patients. The early investigations suggest that all antibodies bind intracellular (self) antigens, with one antibody also exhibiting robust surface binding, supporting the idea of self-reactivity among tumor-infiltrating B cells. Combined, the Spatial VDJ technology developments can facilitate spatially resolved antigen receptor discovery in TLS across cancers and species. Finally, Dr Engblom’s work aims to provide a framework to go from B cell receptor tissue location to antigen discovery (Fig. 1, purple panel).

## Spatial transcriptomics reveals key determinants of response to immunotherapy in patients with NSCLC

Paola Nistico (Head of immunology and immunotherapy unit IRCCS Regina Elena National cancer Institute, Roma, Italy)

Paola Nisticò opened her presentation by sharing recently published findings from her laboratory that shed light on the role of B cells in shaping durable anti-tumor responses in non–small cell lung cancer (NSCLC) patients treated with perioperative chemo-immunocheckpoint inhibitors (ICI) therapy. Using spatial transcriptomics, her group identified a striking enrichment of TLS within the regression bed of the post-treatment primary tumor. Integration of spatial transcriptomics data with scRNA-seq atlases revealed a predominance of activated B cells, memory B cells, and plasma cells in these post-treatment tissues [8].

Interestingly, ongoing analyses of cancer-associated fibroblasts (CAFs) through deconvolution approaches uncovered an enrichment of a specific immune-activating CAF subtype, suggesting a possible contribution of the tumor stroma to the maintenance of immune activation. Although derived from a single case, these observations highlight the potential of B cells and TLS-related biomarkers to inform more precise clinical management in NSCLC [9].

In the second part of her talk, Dr. Nisticò presented unpublished results from a study exploring the determinants of response to adjuvant ICB therapy in NSCLC patients. Through spatial transcriptomic profiling combined with ad-hoc bioinformatic analyses, her team investigated the interplay between TLS and CAF populations across patients with poor, good, and superior responses to therapy. Preliminary data indicate that distinct CAF subtypes (CAF1 and CAF2) exhibit variable spatial proximity to TLS, suggesting that CAF/TLS communication networks may influence the magnitude and quality of anti-tumor immune responses, indicating the tumor microenvironmental architecture as a determinant of clinical outcomes in immunotherapy-treated NSCLC (Fig. 1, yellow panel).

## Spatial organization of immunotherapy responses in TLS and tumor parenchyma

Daniela Thommen (Group leader, division of molecular oncology & Immunology, oncode Institute, Netherlands cancer Institute, Amsterdam, NL)

Recent research emphasizes the role of TLS in mediating response to ICB, but their specific contribution to treatment-induced anti-tumor immunity, and in particular T cell reinvigoration, is not well understood. Daniela Thommen presented results from combining high-dimensional omics analyses with ex vivo technologies to investigate how TLS impacts T cell specificity, state, and capacity for therapeutic reinvigoration in lung tumors. T cell receptor (TCR)*β* sequencing of micro-dissected TLS and tumor areas revealed shared expanded TCRs between those regions, indicating T cell activity in TLS is not driven by a distinct T cell repertoire. Most shared TCRs exhibited dysfunctional phenotypes, suggestive of tumor reactivity. Spatial transcriptomic analyses revealed that T cells in TLS regions were biased towards early dysfunctional states, whereas tumor bed regions enriched for cells with terminally exhausted and cytotoxic phenotypes. To assess reinvigoration capacity, Thommen’s lab exploited their patient-derived tumor fragment ex vivo technology [10] with anti-PD-1 treatment. This demonstrated that tumor-specific T cells in both TLS and tumor parenchyma could be reactivated, but TLS exhibited higher intensity and diversity in cytokine and chemokine secretion. These findings suggest TLS contribute to therapy response by serving as reservoirs for precursor-like cells that may replenish the antitumor T cell pool, and by amplification of T cell responses upon PD-1 blockade (Fig. 1, blue panel).

## B cells and TLS are regulated by immune–tumor–stromal interactions

Tullia C. Bruno (Assistant Professor of Immunology, University of Pittsburgh School of Medicine, Pittsburgh, USA)

B cells are a major component of both the tumor microenvironment (TME) and TLS within solid tumors. Tullia Bruno’s translational research lab utilizes multispectral imaging and spatial transcriptomics to interrogate B cells and TLS in cancer. Indeed, they have demonstrated that the tumor site governs TLS formation and activity in ovarian cancer patients [11]. They developed an in-situ TLS signature that is shared across all three sites of disease (ovary, fallopian tube, and omentum), which correlates with improved prognosis and has unified B cell targets that can be therapeutically tested within physiologically relevant murine models that spontaneously develop TLS. Within this study, they also uncovered the importance of stromal precursors i.e. the mesenchymal stem cells (MSCs) for TLS formation. Specifically, when cancer-educated MSCs (CA-MSCs) are within the ovarian TME, they lead to reduced B cell function and a blunting of MSC differentiation to FDC, which are important for TLS formation. This work was also one of the first published rubrics for the three TLS states—lymphoid aggregate (LA), TLS without a GC and TLS with a GC. They further showed that TLS activity can vary within each TLS state via interrogation of Ki67 (lymphocyte proliferation) and AID (somatic hypermutation of B cells). Specifically, TLS with GC always have higher B/T cell activity. However, in the metastatic setting i.e. brain metastases (mets) all patients form LAs but those with melanoma brain mets have functionally active LAs that correlate with improved prognosis whereas patients with lung brain mets have less active LAs that don’t correlate with improved prognosis.

Tullia Bruno’s lab also studies B cell function outside of a TLS via the extrafollicular (EF) pathway. In autoimmunity and chronic viral infections, B cells are educated via the EF pathway, which can lead to antigen-specific humoral responses. However, they demonstrated that B cells undergoing EF differentiation are less functional than GC-educated B cells [12]. They have termed these B cells “exhausted” as they accumulate in advanced disease, have altered homing receptors, and possess several B-cell-specific inhibitory receptors that blunt B cell function (Fig. 1, brown panel).

## Spatially-determined TLS features associated with resistance to immunotherapy in kidney cancer

Isaias Hernandez-Verdin

(Postdoctorate, Cancer immunotherapy group, Cordeliers Research Centre, INSERM U1138, Paris, France)

TLS are generally associated with improved responses to ICI in several cancers, including clear cell renal cell carcinoma (ccRCC) [2, 3, 13, 14]. Isaias Hernandez addressed the question why many TLS-positive tumors remain resistant to ICI, searching for the presence of tumor-intrinsic immunosuppressive mechanisms.

To investigate this, Isaias Hernandez-Verdin and coll. performed an integrative analysis of ccRCC samples using bulk, single-cell, and spatial transcriptomics, metabolomics, and multiplex immunofluorescence. Comparative transcriptomic analysis of TLS-positive tumors from ICI responders (R) and non-responders (NR) revealed upregulation of four GABA receptor–related genes (GABBR1, GNAI1, ADCY2, and APBA1), defining a “GABA ccRCC signature.” This signature identified a “GABA-high” subgroup with higher NR rates and shorter progression-free survival, validated across three independent cohorts (total *n* = 1,107).

Spatial transcriptomic and metabolic profiling indicated that proximal tubule-like tumor cells produce GABA near immature TLS in NR tumors, leading to impaired immune activation, reduced antigen presentation, and elevated tricarboxylic acid (TCA) cycle activity. Spatial metabolomics confirmed accumulation of TCA cycle intermediates in TLS located near GABA-producing tumor regions. In vitro, GABA inhibited antibody secretion by human B cells, while in vivo, combining anti-PD1 with a GABA synthesis inhibitor (3-MPA) enhanced antitumor efficacy and was further validated in a TLS-rich intraperitoneal model.

Dr Hernandez and his collaborators’ findings identify GABA as a novel immunosuppressive metabolite driving ICI resistance in TLS-positive ccRCC and highlight the therapeutic potential of targeting the GABAergic axis to restore TLS function (Fig. 1, orange panel).

## Concluding remarks

Professor Wolf Herman Fridman concluded the session by highlighting the progress which has been made in the field since the last pre-CICON2023 workshop held in Milano. The positive impact of mature TLS on patients’ response to immunotherapy has been extended to many cancer types. TLS appear not only as reservoirs for precursor-like T cells that replenish the tumor microenvironment but also producing plasma cells that migrate in the tumor bed and secrete antibodies binding to tumor cell antigens. However, not all patients with intratumoral TLS respond to immunotherapy. The burst in high resolution spatial technologies has deepened our understanding of the diversity of TLS states, as well as the impact of intratumoral heterogeneity on TLS formation and function, highlighting the impact of their close neighborhoods on their functionality. The crucial questions of the specificity of T and B cell immune responses activated in TLS are currently addressed, using spatial VDJ technologies. Physiologically relevant murine models that spontaneously develop TLS have been developed, helping to solve the key issue of the muti-steps initiation of TLS formation in cancer. Strategies to induce TLS neogenesis are currently developed, and their use in combination therapies represent promising avenues for cancer treatment.

## Data Availability

Not applicable.

## Abbreviations

TLS: Tertiary lymphoid structures
HES: Hematoxylin, Eosin and Saffron
FDC: Follicular dendritic cells
mTLS: Mature TLS
GC: Germinal center
AAV: Adeno-associated viral
scRNA-seq: Single cell RNA sequencing
NSCLC: Non–small cell lung cancer
ICI: Immunocheckpoint inhibitors
CAFs: Cancer-associated fibroblasts
TCR: T cell receptor
TME: Tumor microenvironment
MSCs: Mesenchymal stem cells
CA-MSCs: Cancer-educated MSCs
mets: Metastases
EF: Extrafollicular
R: Responders
NR: Non-responders
TCA: Tricarboxylic acid
